# Indents

**Published:** 1903-03-15

**Authors:** 


### INDENTS.

☞ **Brief Articles of Technical or Scientific Value will receive notice and comment. Contributions invited.**

**Polishing Proximal Surfaces of the Teeth.** Use rubber bands as carriers and coat them with a mixture of pumice and full strength "*dioxogen.*"—W. A. Mills, in *Cosmos.*

**For Bad Breath.** Use one part of hydrogen dioxid in five parts of rose water, if the malodors come from the mouth. It should be used as a gargle or mouth-wash as often as necessary.

**Polishing Vulcanite Gum Festoons.** Dr. F. H. Smith, in *Dental Brief*, says to cut disks from cork stoppers and mount on the lathe mandrels for polishing the narrow spaces between the teeth.

**Babbitt-metal.** All real babbitt-metal contains copper. To add more, put it in the form of filings in the bottom of the crucible, and the babbitt-metal on top, and cover all with common soda; melt and stir.—*Power and Transmission.*

**For Sensitive Roots.** Apply a saturated solution of carbonate of potassium and glycerine as often as indications require until the sensitiveness disappears. It is effective and does not discolor the root. —Dr. G. W. Johnson in *Dental Review.*

**Courtesy to Physicians.** According to the *British Medical Journal*, as a matter of ethics a medical practitioner has no right to the services of a dentist for either himself or his family gratutiously, or to any reduction of the ordinary fees.—*Dental Record.*

**Soldering Fluid for Gold.**

| | | |
|---|---|---|
| ℞ | Boracic acid, - - - | 1 ounce. |
| | Ammonia carbonate - | 10 grains. |
| | Soda Bicarbonate - - | 1 drachm. |
| | Ammonia water - - | ½ ounce. |

Mix, and shake well before using.—*Dental Clippings.*

**To Fix Points in Socket Handles.** Cover the threaded points with adhesive wax and while hot screw into the socket of the handle which has also been previously warmed. When the wax has cooled the point will be firmly fixed and difficult to remove without heating. The wax also precludes rusting of the joint.—*Office and Laboratory.*

**Bleaching Teeth.** Acetozone is one of the best bleaching agents for discolored dentin that I have ever tried, considering the manipulation required and the results obtained. It will bleach effectively dentin discolored by iodine.

It should be sealed into the tooth from twenty-four to forty-eight hours. If the root-canal is to be bleached, the apex should be filled with chloro-percha, before the Acetozone (powder) is packed into the cavity. Acetozone will penetrate cement, so it should be covered with gutta-percha or other temporary stopping.—*O. E. Lanphear.*

**Pepsin as a Pulp Digestor.** A London, Eng., correspondent of the *Dental Cosmos*, February, 1873, writes that Oakley Cales stated to the Odontological Society of Great Britain, that he had used with considerable success a paste made of *Pepsina Porci* and wo percent hydrochloric acid for digesting the necrotic portion of diseased pulps. The application was sealed into the tooth with cement for three days, when it was found that the necrotic portion had been dissolved and could readily be washed out leaving the living portion of the pulp clean and ready for capping.

Have we lost the art of saving diseased pulps, or have we learned the futility of making the effort? Does this not also suggest that recent methods of digesting pulp tissue were discovered some thirty years ago?

**Two Teeth for One Drunk.** From Constantinople comes a story which should find a place in all future editions of "The Thousand and One Nights:" its grim humor is characteristically Oriental. Abdul, as we all know, is a man of broad views; he acknowledges the right of his subjects to get as drunk as they please in their own homes; but woe-betide them if they are found in a public dramshop. Black-listing is but child's play to the punishment meted out in the land of the Turk. An unfortunate policeman discovered the other day in a state of intoxication was haled before a Pasha, to whom he explained that he had taken a little wine for his tooth's sake. Immediately a barber-surgeon was called in to examine the man's mouth, but no decayed tooth was found. The policeman therefore had to point out the offending molar, which was at once extracted, and handed to the Pasha. That gentleman at once informed the barber that he had taken the wrong tooth, and ordered him to remove the one next to it. This was done, and the poor policeman was dismissed, with the parting shot that his remaining teeth were not likely to ache to the extent of making him forget the imperial prohibition regarding drink.—*Dental Record.*

**Lockjaw Caused by Decayed Teeth.** An inquest was held recently at Bethnal Green, on a young man, a horsehair dresser. The father deposed that his son had worked for a Mr. Pells ever since he was twelve years of age, and had never had a day's illness. His work consisted of combing and dressing horses' manes and tails that came from abroad. A fortnight before Christmas he complained of pains about the body and head. He was attended by Dr. Kempthorne, and appeared to get much better. On Monday, the 22nd inst., he left home in order to see the doctor, having received permission to get up. On the way to the surgery he had a shave, but witness did not see any cut on his face. After his return from the doctor he complained of pain in the shoulders and cheek bones. His jaws became firmly fixed on Tuesday morning and remained so until his death on Christmas Day. The Coroner: It is certain that horses have lockjaw, and the germs may have been in the horsehair. The poison is said also to lie in the earth, but the strange part of this case is that this young man had not the slightest scratch of any kind. Dr. Albert Edward Kempthorne, of Cambribge Road, stated that Smith had influenza and broncho-pneumonia, and appeared to have made a good recovery. When witness was called on the 23rd instant, he found the jaws firmly fixed, and any attempt to administer food or medicine only made the tetanic spasms worse. The autopsy showed that all the organs were congested and pneumonia was present. There was nothing to show the cause of the lockjaw with the exception of the teeth. He concluded that the reflex irritation from the bad teeth might have set up the mischief. The teeth were in very bad condition. He had never had such a case before. The Coroner: It certainly is very mysterious. I suppose we can only say lockjaw caused by bad teeth. The jury returned a verdict of death from lockjaw caused by decayed teeth. —*Dental Record.*

